# The Effect of Traditional Opposition Games on University Students' Mood States: The Score and Group Type as Key Aspects

**DOI:** 10.3389/fpsyg.2020.589323

**Published:** 2021-01-28

**Authors:** María Isabel Cifo Izquierdo, Verónica Alcaraz-Muñoz, Gemma Maria Gea-García, Juan Luis Yuste-Lucas, José Ignacio Alonso Roque

**Affiliations:** ^1^Faculty of Education, Murcia University, Murcia, Spain; ^2^Faculty of Sports, San Antonio Catholic University (UCAM), Murcia, Spain

**Keywords:** physical education, opposition games, motor action, mood swings, POMS

## Abstract

Playing traditional games has a direct impact on the mood states of the players, and this is the reason why physical education is an ideal setting for teaching how to recognize them and be aware about how they can swing. The objective of the study was to determine if participating in traditional opposition games causes changes to the participants' mood states. A total of 102 students participated. Each participant recorded the intensity of the mood state experienced at the beginning and at the end of the sessions, using the validated Profile of Mood States (POMS) instrument. The pedagogical experience was planned as 4 sessions with 6 and 5 opposition games each: (a) with competition in mixed groups, equally and unequally mixed; (b) without competition in mixed groups, equally and unequally mixed; (c) with competition in same-gender groups; and (d) without competition in same-gender groups. When comparing the different mood states according to session (with or without competition), the mood states of the depression, fatigue, and vigor dimensions were significantly different (*p* < 0.05), with higher scores in the sessions with competition for the mood states of vigor (M_competition_ = 7.27 and M_no_competition_ = 3.10) and fatigue (M_competition_ = 4.08 and M_no_competition_ = 1.80). Also, when comparing the mood states depending on session grouping and group type, the results showed differences in the scores obtained for the dimensions fatigue and anger, and general mood state (*p* < 0.05). These differences were found at the start of the session and at the end, with the dimension fatigue being the only one with differences in both situations when comparing the same-gender, equally-mixed, and unequally-mixed groups for the two types of traditional opposition games compared. In addition, after an analysis of the mood states depending on gender was performed, the results and therefore the significant differences found, were very similar to those obtained according to group type. Lastly, it was concluded that the type of group (equal, mixed & same gender), and gender were decisive, causing variations in the mood states of the students. This provides valuable information for teaching professionals about the structuring and organization of PE sessions, aiming to promoting positive motor experiences.

## Introduction

Different studies have confirmed the positive effects of different motor practices on affective well-being (Rovira et al., 2014; Muñoz et al., 2017; Sáez de Ocaríz et al., 2017; Serna et al., 2017; Duran et al., 2019). The emotional processes that are unleashed by different motor practices in Physical Education (from here on PE) classes should be considered, as these sessions provide a good setting for positively influencing the student's mood states. More specifically, playing traditional games has a direct impact on the mood states of the players, and this is the reason why physical education is an ideal setting for teaching how to recognize them and be aware about how they can swing (Parlebas, 2001; Lagardera and Lavega, 2004).

For McNair et al. (1971), mood states (MS) are fluctuating transitory affective states, while for Rubinstein (1981), these are indeterminate general states. However, by taking into consideration the different classifications analyzed, Shuare (1990, p. 77) defines them as “a general and indeterminate mood state of the personality, which influences the cognitive process and behavior with respect to the medium and oneself; it is variable and experiences of differences types, connotation and intensity cohabit within it.” Also, they appear as responses to global assessments about everything that revolves around a subject (Bisquerra, 2000). In MS stimuli are slow and cumulative, but the duration of the responses is long-lasting and pre-dispose cognition, thereby influencing information processing (Gallardo, 2006).

Therefore, the MS can become chronic and established as personality traits (Bisquerra, 2000). Favoring the acquisition of the positive features of personality must be important for every PE teacher who wagers on a modern and innovative perspective. In this sense, a pedagogy of motor conduct would be the basis for any intervention when considering all the personality dimensions of the students: the corporal, the cognitive, the relational, and the affective dimensions (Lagardera and Lavega, 2004). When integrated into the whole of a person, affectivity becomes an indispensable part of motor action (Alonso et al., 2018). Given that affectivity is the key to motricity (Parlebas, 1970), teaching motor conduct in PE classes is indispensable. Considering the internal logic of the motor practices, PE teachers can regulate educational experiences.

In this sense, traditional games can be an ideal medium for regulating education experiences through PE sessions. These games offer a great variety of possibilities of motor actions (Parlebas, 2020), and bring possibilities of emotional self-control.

### Sporting Games and Mood States

There is a correlation between motor practice, MS and well-being, as the different types of motor practices have different effects on the MS, of the participants (Lavega et al., 2011, 2014; Oiarbide et al., 2014; Muñoz et al., 2017; Serna et al., 2017). This indicates that the effect on MS can be different if students participate in motor games than in sports. Therefore, PE should pay close attention to the internal logic of each motor game to promote a state of well-being in the students, given that the type of domain that predominates in PE can determine the mood swing with which the session ends (Lagardera and Lavega, 2011; Lavega et al., 2013a,b, 2014; Muñoz et al., 2017; Serna et al., 2017; Parlebas, 2020). Thus, PE teachers orient the motor practice to well-being, they would guarantee the educational experience in the different types of domains, so that the participants are able to control their emotions through varied motor experiences. In this sense, it would be interesting to understand the effects of traditional games practices on the MS, games that are frequently played as cultural traditions and determined by social entities (Parlebas, 2001). At the same time, it would be interesting to know the effects of these motor actions domains on MS changes.

The evaluation of the MS can be different when some type of interaction exists (sociomotor: opposition, collaboration, collaboration-opposition) as compared to participating without interacting with others (psychomotor). At the same time, when interaction exists, it can be different when this interaction is in opposition, collaboration and/or collaboration-opposition. In an opposition, at least two adversaries face each other to achieve the motor objective, and in collaboration, at least two classmates collaborate to achieve the motor objective. Lastly, in collaboration-opposition at least two classmates collaborate and another one faces them to achieve the same or a different motor objective. Ultimately, both the psychomotor and the sociomotor practices can favor the acquisition of affective well-being (Gelpi et al., 2014; Lavega et al., 2014). In this sense, the different types of motor practices create unequal MS. On the one hand, in the practices where the presence of opposition does not exist (psychomotor and cooperation), positive MS are unleashed, and they are key to the regulation of affectivity (Serna et al., 2017). On the other hand, in cooperation-opposition games, changes in the general MS can occur (Oiarbide et al., 2014), and when opposition is the case, the distress factor increases (Reigal et al., 2013). Thus, it is interesting and important to continue discovering the effects of opposition on the MS of the game participants: traditional, opposition games give more importance to each participant, due to their structural characteristics.

### Competition and Mood States

When participating in motor games, which include the motor communications of cooperation and competition, the intensity of positive MS increases. Meanwhile, in the motor games lacking motor communication or competition, the intensity of negative MS increases (Muñoz et al., 2017). Well-being seems to depend on playing these games and everything else around it (Lavega, 2018). Therefore, aside from the type of practice and domain, another variable that PE teachers should be aware of, as related to the internal logic of the motor game, is the presence or absence of competition. This is defined through the score system and mirrors the achievement of the coded objectives of a sports game (Parlebas, 2001). In this sense, Lavega et al. (2013a) have described the different effects of the presence or absence of competition on MS, and more specifically the affective intensity in the participants caused by the presence of competition. Therefore, positive effects for life in general and co-habiting are found when there is competition, given that the participants learn how to manage victory and defeat (Ames, 1984). Due to this, the PE teachers should also pay attention to the presence (with competition) or not (without competition) of the final score, and understand the effects produced on MS.

In cooperation games, Muñoz et al. (2014) observed high value of “vigor” factor, with competition being a determining factor. In fact, Molina (2016) stated that cooperation games, with or without competition, were excellent pedagogic resources for improving MS. In this study, the players did not show initial differences when playing games with or without competition, however, differences were found at the end of the session with respect to factor fatigue in games with competition. This demonstrates that participation in motor practices determines the emotional state, as in games with competition participants make the maximum effort to win, resulting in a considerable increase in “fatigue” (Oiarbide et al., 2014; Molina, 2016). In psychomotor games, Costes et al. (2014) observed changes in MS. General MS improved, but when the games had a competition factor, a great increase in the MS was produced, which was identified with “vigor.”

### Didactic Logic and Mood Swings

When considering the gender variable, previous studies have provided different conclusions on the experience of MS. When participating in different types of games, boys and girls experience unequal MS (Etxebeste et al., 2014; Muñoz et al., 2017), although they could also experience the same MS (Serna et al., 2017). These differences could be conditioned by the type of motor game, the sporting profile of each participant, or the cultural stereotypes (Muñoz et al., 2017; Serna et al., 2017; Duran et al., 2019); they could also be accentuated depending on the presence or absence of competition (Lavega et al., 2014; Muñoz et al., 2017); ultimately, it is important to promote co-education to guarantee equality to both genders (Torres, 2005; Sáez de Ocaríz et al., 2017).

Similarly, PE teachers should consider other aspects of the internal logic of the motor games that have a direct relationship with the state of well-being, such as the type of group (Muñoz et al., 2017; Serna et al., 2017; Duran et al., 2019). When discussing the type of group, we can differentiate between mixed groups (groups composed of students of different gender) and segregated groups (groups composed by students of the same gender), as gender determines the preference for the type of practice (Etxebeste, 2012). On the one hand, when participating in expressive psychomotor situations in segregated groups (same gender groups), girls perceived the MS of “anger-hostility” with less intensity than boys; and when participating in mixed groups the intensity of the “vigor-activity” factor increased for both genders (Duran et al., 2019). On the other hand, when participating in psychomotor or sociomotor games of cooperation in a segregated group, a greater intensity was observed for the “fatigue-immobility” factor when there was no interaction, as compared to when the participants cooperated, while no differences were observed when participating in mixed groups (Muñoz et al., 2017). Therefore, the didactic logic of the educators will also be a determinant factor in promoting a modern PE class, as previous studies have shown the possibilities offered by group organization into mixed groups for promoting the experience of positive mood swings and the reduction of the negative ones (Solmon, 1996; Lavega et al., 2014; Muñoz et al., 2017).

Given that traditional games offer unique structural possibilities of opposition that grant more importance to the players, it is necessary to know what changes in their MS are produce. Therefore, the main objectives of the study were: (a) To analyze what occurs with students' MS when they participate in sessions of traditional opposition games; (b) To determine what occurs as a function of the presence or absence of competition in these traditional games and lastly; and (c) To determine how the gender configuration of group influences the levels of activation of the MS during the traditional opposition games with or without competition.

## Materials and Methods

### Design and Participants

A descriptive cross-sectional method was utilized, with a pretest-posttest design with different samples. The selection of the sample was performed through non-probabilistic convenience sampling (Hernández-Sampieri et al., 2010). A total of 102 university students participated, with a mean age of 20.14 ± 1.48. Of these, 78 of them were men [20.26 ± 1.6 years old (76.5%)] and 24 were women [19.75 ± 0.94 years old (23.5%)]. For conducting the sessions, the sample was divided into three groups as a function of gender, in the following manner: same group, composed entirely by 24 men (M = 20.17 ± 0.92 years old); equally-mixed, composed by a total of 21 participants, both men and women (N_women_ = 11, N_men_ = 10, M = 20.29 ± 1.52 years old); and an unequally-mixed group (N_women_ = 12, N_men_ = 45, M = 20.07 ± 1.67 years old).

### Measurements and Materials

#### Profile of Mood State Questionnaire

The participants completed the Spanish version of the questionnaire Profile of Mood States (POMS) from McNair et al. (1971), in its reduced, adapted, and validated form (Fuentes et al., 1995). This version of POMS questionnaire has a total of 29 items grouped into five factors (Balaguer et al., 1993; Arruza et al., 1998). “Tension-Anxiety” (TA) (6 items): tense, restless, uneasy, nervous, anxious, restful. “Depression-Dejection” (DD), (6 items): helpless, sad, unhappy, discouraged, unfortunate, bitter. “Anger-Hostility” (AH) (6 items): angry, grouchy, annoyed, furious, on-edge, resentful. “Vigor-Activity” (VA) (6 items): energetic, lively, vigorous, full of pep, active, hardy.And “Fatigue-Inertia” (FI) (5 items): bushed, exhausted, fatigued, worn out, tired. The score for each of the items was recorded with a Likert-type scale ranging from 0 to 4, where 0 = “not at all” to 4 = “high intensity or extremely.” For each identified factor a mean value of MS intensity experienced was obtained. Through the sum of all the scores obtained, this instrument allowed obtaining a generic score for each factors. Lastly, an overall score of the general mood state (GMS) was obtained with Equation (1) (McNair et al., 1971). This GMS score was calculated based on the establishment of an initial value of 100, to which the score found for the dimension VA (defined as a positive dimension) is added to, with the scores found for the other dimensions subtracted from it (TA, DD, AH and FI, considered as negative). This instrument was shown to have the same psychometric quality as the original version with 58 adjectives, with the Cronbach's Alpha index for each of the dimensions ranging from 0.78 to 0.83.

(1) $$\begin{array}{r} \text{GMS}=100+\text{VA}-\text{TA}-\text{DD}-\text{AH}-\text{FI} \end{array}$$

#### Traditional Opposition Game Sessions

In order to select the games to be play, the following procedure was followed: (a) a review of the existing literature related to traditional motor games; (b) identification of traditional sports games in Spain without a gender bias; (c) determination and selection of traditional motor games BELONGEN TO the motor domain of opposition; (d) selection of traditional sports games that could be implemented in standardized sport spaces; (e) selection of traditional motor games not require an excessive amount of sports equipment; and (f) selection of games that are easy to play.

In relation to the selection procedure, traditional games are classified according to the interactions between players. The opposition domain is a domain in which a player faces at least one other player. In this type of motor situations, the participants share a language of confusing signs, of messages that hide their true intentions in the game, in order to deceive their adversary (Parlebas, 2001; Lavega et al., 2014). On the other hand, traditional motor games were also classified according to a categorization based on the possibility or not of obtaining victory within the practical motor situation developed (Parlebas, 2001). This allows differentiating between two types of game sessions: (a) sessions with competition or competitive games (SCG). Those traditional motor games were identified with the presence of a final score or result within the game, which allows the player to obtain or not victory at the end of the practical situation. As a result, players are classified as winning or losing; (b) sessions without competition or non-competitive games (SWC). Those traditional motor games do not have a final score. Therefore, there is no final score that classifies the players based on obtaining victory or not within motor practice, as they are not based in competition (Parlebas, 2001; Etxebeste et al., 2014; Gea et al., 2016).

Four practical sessions were conducted, with the first and third sessions corresponding with SCG, while the second and fourth sessions included SWC. In the first session, the university students participated in the following traditional opposition games: Black and white, Chinese soccer, Standoff, Pepe captures you, and Coconuts. In the second session, the traditional opposition games were: Copy-Chase, Gone with the wind, Hare to the wall, The clap, and Land, sea and air. Then, the third session was composed by the games: Tail thief, The handkerchief, Touch, Raspall, Musical chairs and Capture with ball. Finally, in the fourth session, the traditional opposition games played were: The princess, Take the broom, The mill, The labyrinth, The earthquake, and Letter envelope. All of these traditional games are well-known and commonly used in PE classes in Spain. An example of some of the traditional opposition games is shown in Table 1. The traditional opposition games were developed following the rules as stated by Lavega and Olaso (1999). Each of these sessions had an average duration of 90 min, and was conducted in the same sports space and at a similar hour which was agreed upon with the participants. The sessions were separated by a period of 7 days.

**Table 1:** Description of the traditional opposition games used in the study.

**Traditional opposition games**				
	**Description**	**Network**		**Score system**
		**Motor communication**	**Sociomotor roles**	
**SCG**				
Standoff (Session 1)	Each player places a foot forward and alongside one of the opponent's feet. After this, both players take each other's hands and try to push the opponent off balance, without their own feet moving. When a player's foot moves, a point is awarded to the other player. The winner is the first player to achieve 5 points.	Exclusive and stable.	Symmetric duel.	With Memory. Score limited.
Black and White (Session 1)	In pairs, face to face, placed one meter from each other. Each player is assigned a name (black or white). The teacher says one of the two names at random. The named player tries to catch the adversary before he or she reaches the end of the track. The winner is the one who catches the other player or escapes without being caught more times.	Exclusive and stable.	Symmetric duel.	With Memory. Score limited.
Tail thief (Session 3)	Each player has a handkerchief or piece of cloth that is placed on the back of the pants. The objective is to get as many handkerchiefs from the other players. The one with the most wins.	Exclusive and stable.	Dymmetric duel.	With Memory. Time limited.
Touch (Session 3)	Two players challenge each other to try to touch the posterior part of the adversary's body (shoulder, back, legs, etc.) as many times as possible. The winner is the first player to achieve 5 points.	Exclusive and stable.	Symmetric duel.	With Memory. Score limited.
**SWC**				
Copy-Chase (Session 2)	One participant moves around the playground in whatever way he/she wishes. The others players must copy the first player's movements. The first player must catch one of the restof the players, and then they switch roles.	Exclusive and unstable.	Permutation.	Without memory.
Gone with the wind (Session 2)	All participants except one are sitting in a circle. One participant is placed in the center of this circle. That player must say “it is so windy that it takes away everyone who wearing a.” At this moment, all the players who comply with that requirement must get up and change their place. The player who is left without a place goes to the middle to give the next instruction.	Exclusive and unstable.	Permutation.	Without memory.
The princess (Session 4)	Groups of 5 in a circle. One of the participants plays the role of the prince or guardian, while the rest are thieves. A cone or similar object, which will the “princess” is placed in the center of the circle. The prince or guardian will try to protect the princess from the “touch” of the thieves.	Exclusive and unstable.	Permutation.	Without memory.
The labyrinth (Session 4)	In a large group. The players move about the field trying to immobilize the rest of the participants. The way to immobilize is through touching or contact with the right forearm of the player. Afterwards, the player is immobilized (knee to the ground).	Exclusive and stable.	Disymmetric duel.	Without memory.

*SCG, sessions with competition games. In this case, each player competes against an adversary; SWC, sessions with games without competition. There is no winner or loser at the end of the game. Results are not compared. Players exchange roles during the game*.

Lastly, the SCG were composed by a total of 11 different games, divided into five games in the first session and six games in the fourth session. In the case of the SWC, the total number of the games was also 11, divided into five games in the third session and six games in the fourth session.

### Procedures

#### Previous Information and Informed Consent

The following protocol was established to guarantee a similar collection of data in each and every session during the study. In first place, students enrolled in the 2nd year of Physical Education and Sports Science (PESS) Degree were contacted.

After students accepted to participate in the study, a second meeting was conducted. On this occasion, they were provided with all the documentation necessary for them to understand the reasons and benefits for their future teaching practice provided by these interventions within the education sphere. In this meeting, the researchers explained the process that would take place and responded to all the doubts that emerged related to the measurement protocol to be followed. At the same time, an adequate schedule was agreed upon with the participants that was similar with respect to the days and the times for the sessions. Afterwards, the participants signed a voluntary participation informed consent form. The study was conducted in agreement with the ethical principle of the Declaration of Helsinki for human research (World Medical Association, 2013), and was approved by the institutional review boards of the participating Universities.

#### Initial Familiarization and Distribution of Groups

In first place, all the participants attended a session of practical training that lasted 1 h. In this session, the participants received training on emotional education, in order to correctly identify the MS experienced at the theoretical, as well as the practical level. In this same session, a practical simulation was conducted that allowed the participants to familiarize themselves with the POMS questionnaire that would be used posteriorly in the practical sessions (Lavega et al., 2011, 2013a,b, 2014; Gelpi et al., 2014; Gea et al., 2016; Muñoz et al., 2017).

As for the creation of the intervention groups, three types of groups were established for the intervention. The first group was a same group (unmixed). In the second group, the intervention was conducted with an equally-mixed group (mixed), where both genders participated (males and females), and with the number of participants being the same for both genders. Lastly, in the third group, the intervention was conducted with an unequal-mixed group (unequal). This was also comprised by students of different genders, with the number of participants according to gender being unequal.

In order to carry out the different sessions of traditional games, the groups and the participants remained the same in the all sessions.

#### Measurements

Before starting each session, each participant was provided with a booklet that contained the POMS questionnaire. Although the participants were already familiarized with the questionnaire, the teachers proceeded to briefly recall the structure and sections to fill out at the beginning of each of the sessions. The university students proceeded to fill in the questionnaire at the beginning the session and immediately at the end of each the sessions. For the collection of data through the POMS questionnaire, each student proceeded to complete the questionnaire individually and in silence. Finally, to calculate a total score obtained in each session, grouped according to their categorization, which was based on the possibility or not of obtaining victory, the average value was calculated for each MS. The total resulting scores were named as follows: Competition_T (average value sessions 1 and 3), and No_Competition_T (average value sessions 2 and 4).

The same teacher directed each and every session. To homogenize the playing of the games conditions in all the sessions, the teacher always gave the same instructions prior to the start of the sessions. Likewise, the games were explained to the participants in such a manner that the students were not encouraged, exhorted or motivated during any of the different games played.

### Statistical Analysis

The descriptive data for the different variables of the study are shown as the mean and standard deviation. The Kolmogorov-Smirnov test was utilized to verify data normality. Data did not have an homogeneous distribution. Hence, non-parametric tests were applied in the statistical analysis. To detect the differences in MS scores as a function of the session and moment in which the questionnaire was given (prior to the start or after the end of the session), the data were analyzed with the non-parametric Friedman test, with the *post-hoc* Wilcoxon test for pair-wise comparisons. Lastly, for studying the variations in intensity shown in the MS as a function of the group type and gender, the non-parametric Kruskal-Wallis test was utilized, together with the *post-hoc* non-parametric Mann-Whitney *U-*test for posterior pair-wise comparisons in the cases where this was necessary. Effect size was calculated with Rosenthal's *r* (Rosenthal, 1991; Lenhard and Lenhard, 2016) and η^2^ (Tomczak and Tomcak, 2014) [0.1–0.3 (small); 0.3–0.5 (medium) and >0.5 (large) effect]. A significance level of *p* < 0.05 was accepted for statistical comparisons, except in the case of the multiple comparisons a posteriori for the comparison of independent variables, where the significance level was set at *p* < 0.008. All calculations were performed with SPSS Statistics for Windows, Version 24.0 (IBM Corp., Armonk, NY).

## Results

**Figure 1:**
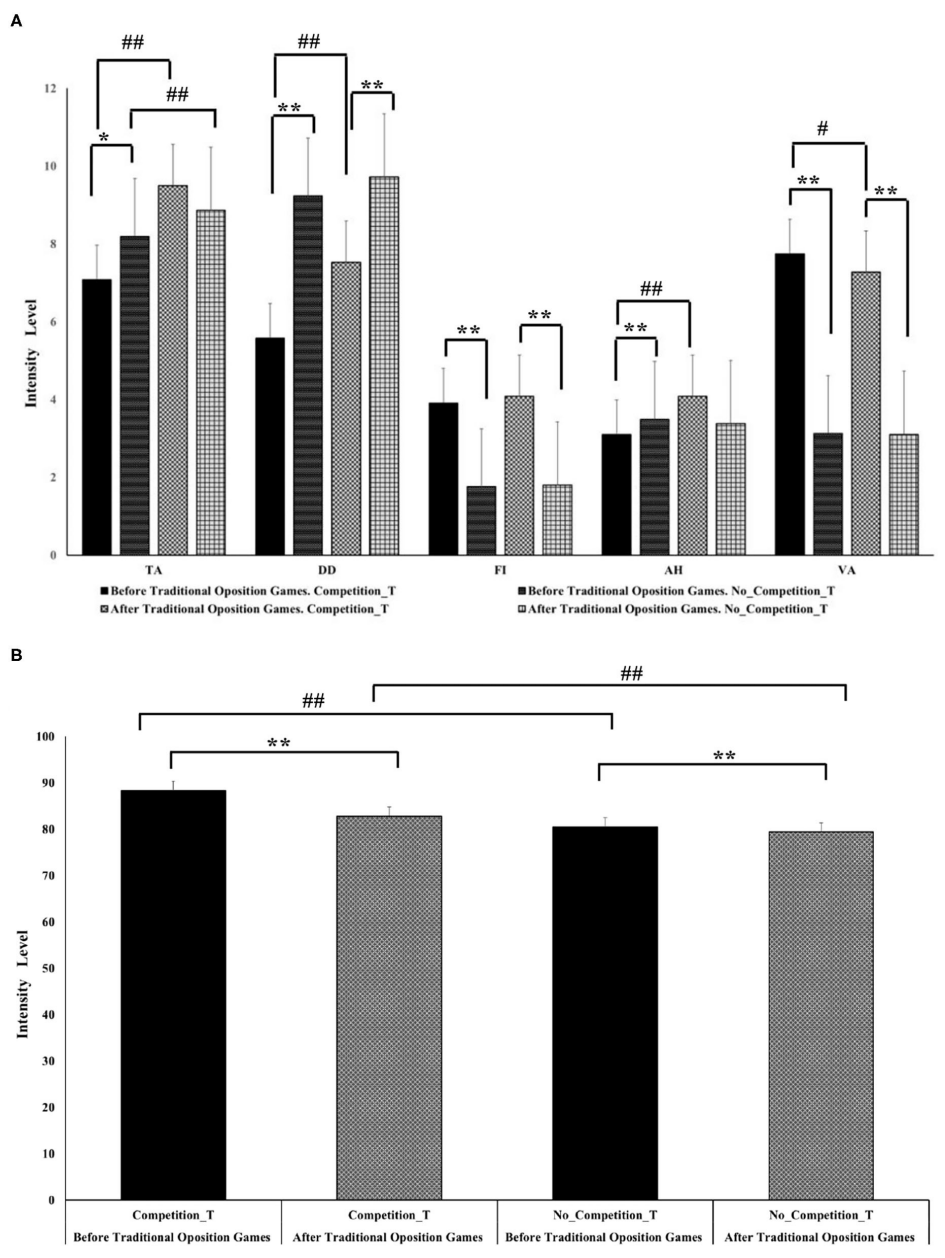
Recorded intensity for individual and general score of mood states in each type of traditional sport games session. **(A)** Intensity recorded per session in the mood states. **(B)** Intensity recorded per session for the overall score of the general mood state. Error bars indicate standard error. * = significant difference between the score of mood states at every step of the measurements. *p* < 0.05. # = significant difference between the score of mood states between the pairwise before and after the intervention. *p* < 0.05. ** = *p* < 0.01, ## = *p* < 0.01.

As shown in Figure 1A, the different dimensions that comprise the POMS scale obtained intensity values that were significantly different before the start of each of the sessions.

More specifically, significant differences were found when comparing the intensity described by the university students for the Competition_T and No_Competition_T score for all the MS dimensions before participating in the game sessions (*p* < 0.05). For the TA (χ^2^ = 9.91, *r* = 0.42), DD (χ^2^ = 70.04, *r* = 0.79), and AH (χ^2^ = 12.23, *r* = 0.27) dimensions, the levels of activation recorded for the MS were lower in the average competition total score. On the other hand, for the rest of the dimensions [FI (χ^2^ = 40.05, *r* = 0.59) and VA (χ^2^ = 69.18, *r* = 0.78)], the results showed higher scores prior to the sessions. Similar results were found in the GMS, obtaining the highest general score of activation in the competition total score (χ^2^ = 45.37, *r* = 0.73) (Figure 1B).

Next, when comparing the scores obtained for the MS after the interventions with traditional sports games sessions, significant differences were found in DD, FI, and VA dimensions, as well as GMS (*p* < 0.05) (Figure 1). More precisely, the FI (χ^2^ = 53.06, *r* = 0.67), VA (χ^2^ = 77.44, *r* = 0.84), and GMS (χ^2^ = 23.77, *r* = 0.49) total scores increased after the competition traditional sports games, while the DD dimension total score increased after the no_competition traditional sports games (χ^2^ = 42.67, *r* = 0.65).

Finally, when pairwise comparisons were made, the POMS scores showed significant differences between TA (χ^2^ = 48.23, *r* = 0.65, *p* = 0.000), DD (χ^2^ = 45.43, *r* = 0.67, *p* = 0.000), AH (χ^2^ = 11.46, *r* = 0.37, *p* = 0.001), VA (χ^2^ = 4.64, *r* = 0.17, *p* = 0.031), and GMS (χ^2^ = 40.09, *r* = 0.64, *p* = 0.000) before and after the implementation of traditional sports games with competition. Whereas, for the implementation of traditional sports games without competition, significant differences were only found in the TA dimension (χ^2^ = 11.38, *r* = 0.32, *p* = 0.001) and GMS (χ^2^ = 10.67, *r* = 0.27, *p* = 0.001) (Figure 1).

On the other hand, Table 2 shows the significant differences found for all the levels of intensity recorded for each of the dimensions that compose the MS as a function of type of session (competition and no_competition), and type of group.

**Table 2:** Intensity registered as a function of the type of group for the different dimensions of the mood states.

**Mood states recorded according to dimension for each of the sessions as a function of the type of group**														
	**Before traditional opposition games session**							**After traditional opposition games session**						
	**Same gender**		**Equal mixed**		**Unequal mixed**		***p***	**Same gender**		**Equal mixed**		**Unequal mixed**		***p***
	**M**	**SD**	**M**	**SD**	**M**	**SD**		**M**	**SD**	**M**	**SD**	**M**	**SD**	
TA_Competition_T	6.63	2.25	7.48	1.90	7.12	2.68	0.319	9.27	4.22	9.29	2.84	9.84	3.71	0.564
TA_No_Competition_T	8.15	2.66	8.30	2.03	8.17	2.76	0.948	9.04	2.94	9.90	1.93	8.49	3.00	0.110
DD_Competition_T	5.65	2.89	5.02	2.01	5.75	2.90	0.577	8.06	3.31	7.12	2.73	7.46	2.99	0.804
DD_No_Competition_T	9.06	2.90	8.83	1.99	9.45	2.98	0.488	10.19	3.32	10.38	2.61	9.29	3.22	0.396
FI_Competition_T	4.83^b^	3.78	1.98^a^^,^^c^	2.40	4.24^b^	3.88	0.017^*^	5.06^b^	3.41	2.43^a^	1.39	4.28	3.30	0.034^*^
FI_No_Competition_T	1.92	2.83	0.59^c^	1.59	2.12^b^	3.12	0.046^*^	2.69^b^	3.42	0.21^a^^,^^c^	0.66	2.02^b^	3.01	0.004^**^
AH_Competition_T	3.21	2.63	1.57	1.32	3.62	3.54	0.050	4.08^b^	2.90	2.55^a^	1.53	3.82	3.33	0.034^*^
AH_No_Competition_T	4.04	2.99	3.12	1.79	3.40	2.72	0.549	3.96	2.80	2.33	1.95	3.52	2.63	0.077
VA_Competition_T	6.33	2.80	8.31	3.45	8.13	2.94	0.050	7.04	4.29	7.31	2.88	7.36	3.54	0.820
VA_No_Competition_T	3.25	3.36	3.00	2.67	3.12	3.37	0.948	3.31	3.96	1.95	1.96	3.43	3.28	0.165
GMS_Competition_T	86.3^b^	7.25	92.26^a^	5.98	87.73	9.15	0.014^*^	80.56	7.38	85.93	4.94	81.96	9.06	0.051
GMS_No_Competition_T	80.08	6.53	82.30	3.89	79.97	6.88	0.256	77.44	7.32	79.12	4.27	80.11	7.12	0.919

TA, tension-anxiety; DD, depression-dejection; FI, fatigue-inertia; AH, anger-hostility; VA, vigor-activity; GMS, general mood state; Competition_T: average value for scores obtained in sessions 1 and 3; No_Competition: average value for scores obtained in sessions 2 and 4; significant post-hoc differences between groups:

a same gender,

b equal mixed,

c *unequal mixed*.

** p < 0.01;

* *p < 0.05*.

More specifically, before the start of the sessions, significant differences were only observed for the F1 dimension in both types of session (*p* < 0.05, $\eta_{\text{SCG}}^{2}$ = 0.09, $\eta_{\text{SWC}}^{2}$ = 0.04), and in the GMS for the competition total score (*p* < 0.05, $\eta_{\text{SCG}}^{2}$ = 0.05). In the pairwise comparison, it was observed that the score obtained for the FI dimension was lower and significantly different for the equally-mixed group as compared to the other two groups. On the other hand, when comparing the type of group after the end of each type of session, differences were observed for both cases in the scores obtained for the MS in the FI dimension (*p* < 0.05, $\eta_{\text{SCG}}^{2}$ = 0.09, $\eta_{\text{SWC}}^{2}$ = 0.09). More specifically, in the case of games with competition, the pairwise comparisons showed significant differences between the same-gender, equally-mixed groups, with the resulting score being much higher for the same-gender group. On the other hand, in relation to the GMS, the pairwise comparisons showed differences between the scores obtained by the same-gender and equally mixed groups, with a lower score found for the same-gender group.

Lastly, when referring to the games without competition, significant differences were only found for the AH dimension after the end of the different sessions of traditional games of opposition (*p* < 0.05, $\eta_{\text{SCG}}^{2}$ = 0.06). More specifically, when comparing the same-gender and equally-mixed groups, the resulting score for this dimension obtained a higher value in the same-gender group.

The results obtained as a function of the gender of the participants for the scores obtained in the different dimensions of the MS will be analyzed as a group (Table 3).

**Table 3:** Intensity registered as a function of the gender for the different dimensions of the mood states.

**Mood states recorded according to dimension for each of the sessions as a function of the gender**												
	**Before traditional opposition games' session**						**After traditional opposition games' session**					
	**Male**		**Female**		***r***	***P***	**Male**		**Female**		***r***	***p***
	**M**	**SD**	**M**	**SD**			**M**	**SD**	**M**	**SD**		
TA_Competition_T	7.07	2.59	7.11	1.87	0.027	0.789	9.69	3.95	9.27	2.50	0.025	0.800
TA_No_Competition_T	8.21	2.72	8.11	2.10	0.012	0.906	8.82	2.90	9.21	2.65	0.073	0.462
DD_Competition_T	5.58	2.73	5.58	2.80	0.032	0.743	7.59	3.13	7.33	2.62	0.032	0.743
DD_No_Competition_T	9.38	2.77	8.73	2.77	0.115	0.245	9.76	3.17	9.60	3.10	0.053	0.594
FI_Competition_T	4.31	3.73	2.60	3.44	0.222	0.019^*^	4.39	3.30	3.08	2.34	0.168	0.090
FI_No_Competition_T	2.10	3.05	0.65	1.63	0.296	0.003^*^	2.21	3.16	0.48	1.26	0.308	0.002^*^
AH_Competition_T	3.25	3.05	2.63	3.21	0.129	0.194	3.70	2.93	3.38	3.15	0.056	0.547
AH_No_Competition_T	3.62	2.74	3.08	2.21	0.078	0.431	3.55	2.73	2.81	1.97	0.092	0.352
VA_Competition_T	7.64	3.02	8.08	3.37	0.047	0.638	7.31	3.81	7.15	2.73	0.020	0.984
VA_No_Competition_T	3.29	3.31	2.60	2.87	0.100	0.310	3.17	3.46	2.88	2.58	0.018	0.852
GMS_Competition_T	87.54	8.54	91.09	7.22	0.202	0.043^*^	81.94	8.26	84.08	7.67	0.012	0.117
GMS_No_Competition_T	79.97	6.57	82.13	5.23	0.116	0.108	78.82	6.97	80.77	5.71	0.132	0.182

TA, tension-anxiety; DD, depression-dejection; FI, fatigue-inertia; AH, anger-hostility; VA, vigor-activity; GMS, general mood state; Competition_T, average value for scores obtained in sessions 1 and 3; No_Competition, average value for scores obtained in sessions 2 and 4; **p < 0.01;

* *p < 0.05*.

When comparing the intensities recorded for the MS as a function of gender, before the sessions, significant differences were only found in the case of the average of the competition total score for the FI dimension and GMS (*p* < 0.05). For the games with competition, as well as those without competition, the male participants obtained higher scores for the FI dimension, as compared to the female participants (*p* < 0.05). However, for the GMS score, the differences according to gender were only observed for the games with competition session, with the score obtained being higher for the female participants. Lastly, once the sessions had ended, significant differences were only found for the FI dimension in the average of the no-competition total score (*p* < 0.05). In this specific case, the male participants obtained higher scores for the FI dimension than the female participants.

## Discussion

This study was conducted with the intention of discovering how traditional opposition sports games could influence the MS experienced by the PESS university students when they arrive to class, as a function of the presence of competition or not and the type of group.

In first place, before the start of each of the sessions, it was observed that there was a great variability in the intensity recorded for each dimension. At the start of each session, for the different dimensions of the POMS, significantly different intensities were recorded. When comparing the intensity experienced by the university students for the dimensions TA, DD, and AH, lower scores were observed in the session with competition. However, related to dimensions FI, VA, and GMS, higher scores were observed in the session with competition. Therefore, the changes in MS are not only determined by the type of motor activity, but also by the physiological aspects and the subjective perceptions of well-being possessed by each player (Reigal et al., 2013). In this sense, although there was a correlation between motor activity and mood swing, more research is needed to find the cause for this (Balaguer et al., 1993). It is believed that the cause could be that the good MS experienced makes the students want to participate in motor activities and thus improve their well-being, but also that the motor activities make them be in a good MS and therefore have an improved well-being.

Subsequently, in the scores obtained in each of the final sessions, it was observed that the intensity of the positive AV mood increased with the presence of competition in traditional opposition games. This was also found in the study by Muñoz et al. (2017): when the students participated in psychomotor and cooperative games the intensity of the positive mood swings only increased when there was competition. In the case of cooperation games, Muñoz et al. (2014) also observed an increase in VA dimension, with the presence or not of competition being a determining factor. Just as Costes et al. (2014), it was also observed that the presence of competition created and increased MS related with VA. On the other hand, it was observed that the intensity of negative FI factor increased with the presence of competition and decreased with its absence. The same result was found in the work by Molina (2016), as the players showed an increase in FI dimension in competition games.

Significant differences were found for all the dimensions of the MS (except for dimension FI), as well as for GMS scores. When comparing the MS scores obtained before and after the intervention, in the dimensions TA, AH, and DD, higher intensities were recorded at the end of the sessions with competition. These results agree with those by Reigal and Videra (2013), who stated that the students, feelings of depression, anxiety and confusion increased after a session of physical activity. This could be due to the fact that in opposition games, there is interaction with other participants (Lagardera and Lavega, 2004) the achievement of the objective, victory or defeat, does not depend on oneself, so that there could be greater demands in the game that result in the increase of these MS. Perhaps having to play against an adversary does not allow the participants in opposition games to decrease their states of tension and hostility. In any case, as these were different motor activities, it would be necessary to continue research along this line to understand the general MS in each domain (Lavega et al., 2011).

As other research studies contribute, these differences could be due to the characteristics of the internal logic of the game and the subjective logic of the participants (Parlebas, 2001; Muñoz et al., 2017). On the one hand, the internal logic of the game demonstrates that PE intervention determines the mood swing, because in games with competition the participants make the greatest effort to win, resulting in a considerable increase in fatigue (Oiarbide et al., 2014; Molina, 2016). On the other hand, the subjective logic of the participants could be the cause of the students experiencing the MS with a lesser intensity when participating without competition. As these were PESS students, they could have a sports background highly related to competition (Lavega et al., 2011; Muñoz et al., 2017; Duran et al., 2019). Thus, if PE teachers intend to have a positive influence on the mood swings of the students, they should consider the characteristics of the motor activity, as well as the sports background of each student (Muñoz et al., 2017). The results presented provide evidence on the importance of PE teachers when offering a wide range of traditional motor games to the students. A great variety of motor and affective experiences can allow the students to be more prepared in their later adult life. Also, they can allow for consolidating or re-orienting affective experiences as a function of the planned motor situations and their relationship with their sports background.

In this study GMS score decreased independently of the presence or absence of competition. These results coincide with those obtained by Costes et al. (2014), given that they demonstrate that students increased their MS factors when not competing in psychomotor games. Although both studies were conducted with PESS university students, the motor conditions were different. Perhaps this is why not much variability was observed in the results from the Costes et al. (2014) study, as opposed to the study presented herein. In the present work, sociomotor situations of opposition were planned, and therefore the variations in the MS were not dependent only on the person itself, as in the case of the psychomotor situations, but on the presence of adversaries as well.

Another finding related to the traditional opposition sports games was that they type of group that participated in the SCG determined the experience of the MS in the FI dimension. When the group was the same-gender group, FI increased. However, in VA the type of group did not determine changes in the MS of the participants. This was not observed in the study by Muñoz et al. (2017), as the authors attested that there was an increase in intensity in the VA dimension when participating in same-gender groups with respect to mixed groups. These changes in MS could be due to the level of physical conditioning of the participants. Physical conditioning could be different in males and females, so it could determine the intensity when participating in an equally-mixed group (Torres, 2005). Costes et al. (2014) stated that VA factor was the only which increased when competition games were played. As opposed to this, in this study, the results show that the type of group does not determine VA dimension. The difference in results could be because the games played in Costes et al. (2014) were psychomotor in nature, and perhaps the mood swing was exclusively dependent on the participating individual. However, in opposition games the MS can be affected by the motor conduct of the adversary. When playing against an adversary it is necessary to continuously decode the intentions of the other, while at the same time thinking about possible strategies to stop or avoid the adversary, and therefore achieve the objective. This must also be performed quickly to anticipate any response or behavior from the other player.

Thus, if the intention is to reduce the feeling or experience of negative MS when participating in opposition sports games, PE teachers should wager on a mixed group. This highlights the importance of co-education when planning games that increase the positive effects on the MS of the participants (Lavega et al., 2014; Muñoz et al., 2017). In this sense, gender must be an aspect to consider, as the male participants increased FI value with the presence and absence of competition when participating in traditional games of opposition, to guarantee the equality of both genders (Torres, 2005; Sáez de Ocaríz et al., 2017).

## Conclusions

Although the MS were highly variable, it can be confirmed that with experience, the students learned to be aware of the abilities and limitations of the game in affective education. Also, it allowed them to understand and evaluate the changes in their MS if they won or lost, and if they achieved the objective or not (Costes et al., 2014). Oiarbide et al. (2014) affirm that the mood swings depend on everyday processes and the situations experienced modify the MS, and therefore, the internal logic of the different domains in the PE classes could modify the MS of the students. Each study participant could extrapolate his or her experience to the PE classes once they become teachers, with the aim of building a road toward well-being and happiness, although this should be further studied. If future PE teachers understand the benefits of traditional motor games through their personal experience, their awareness is made possible in our society, through their practice in the area of education.

Lastly, the conclusions extracted, which define the findings based on the previously-posed objectives, are:

- Traditional games of opposition offer a great variety of on emotional experiences. The experience of future PE teachers can result in the re-orientation of planned motor situation.
- Traditional games of opposition with competition increase the “tension-anxiety,” “depression-dejection,” and “anger-hostility” MS. Therefore, PE teachers should consider the internal logic of practices to guide the experience toward positive states. The score of the games is key to improving mood swings.
- Participate in mixed groups can reduce the emotional intensity of negative MS in traditional games of opposition. For this reason, the PE teachers should have in mind the didactic logic when designing social motor games.

This study provides valuable information for professional educators about the structure and organization of PE sessions which aim to promote positive motor experiences. If future PE teachers reflect on the practice through their own experience, they could make changes that are guided toward emotional PE.

The main limitation of this study was the profile of the participating students. As the sample was not as representative of the general population, the results cannot be extrapolated to the Compulsory Education stages. Another limitation was the exclusive use of a quantitative research design. A qualitative research design could have allowed external validity, allowing for the generalization of the results, as the study aimed to identify the profound nature of the realities experienced. If both quantitative and qualitative methods had been used, perhaps the biases from the quantitative procedure could have been corrected. Aside from the qualitative analysis of the MS of the students, it would have been interesting to know and understand what this experience meant for the participants, to complement these results and improve future lines of research.

## Data Availability Statement

The raw data supporting the conclusions of this article will be made available by the authors, without undue reservation.

## Ethics Statement

The studies involving human participants were reviewed and approved by The University Ethics Committee of Murcia University reviewed and approved the research in accordance with the principles set out in the Declaration of Helsinki. The patients/participants provided their written informed consent to participate in this study.

## Author Contributions

MC and JA conceptualized and designed the study. GG-G, JY, and VA-M recruited the subjects. GG-G, JA, and VA-M collected the data. JA and MC organized the database. JY and GG-G carried out the statistical analysis. MC, JA, GG-G, VA-M, and JY wrote the first manuscript draft. MC and GG-G developed the final manuscript draft, the English proofreading, reviewed, and edited the final version of the manuscript. All authors contributed to the manuscript revision and approved the definitive manuscript.

## Conflict of Interest

The authors declare that the research was conducted in the absence of any commercial or financial relationships that could be construed as a potential conflict of interest.
